# Serial 12-Lead Electrocardiogram–Based Deep-Learning Model for Hospital Admission Prediction in Emergency Department Cardiac Presentations: Retrospective Cohort Study

**DOI:** 10.2196/80569

**Published:** 2025-10-17

**Authors:** Arda Altintepe, Kutsev Bengisu Ozyoruk

**Affiliations:** 1 Horace Mann School, New York, NY, United States; 2 Department of Radiology and Imaging Sciences, Emory University School of Medicine, Health Sciences Research Building II (HSRB II), 1760 Haygood Dr NE Suite N647, Atlanta, GA, 30322, United States, 1 8573125101, 1 4047125005

**Keywords:** emergency department, electrocardiography, deep learning, multimodal machine learning, transfer learning, hospital admission prediction, cardiac presentations, serial ECG, MIMIC-IV, electrocardiogram

## Abstract

**Background:**

Emergency department (ED) crowding is often attributed to a slow hospitalization process, leading to reduced quality of care. Predicting early disposition in patients presenting with cardiac issues is challenging: most are ultimately discharged, yet those with a cardiac etiology frequently require hospital admission. Existing scores rely on single-time-point data and often underperform when patient risk evolves during the visit.

**Objective:**

This study aimed to develop and validate a real-time deep-learning model that fuses serial 12-lead electrocardiogram (ECG) waveforms with sequential vitals and routinely available clinical data to predict hospital admission early in ED encounters.

**Methods:**

We conducted a retrospective cohort study using the Medical Information Mart for Intensive Care (MIMIC) IV, MIMIC-IV Emergency Department module, and MIMIC-IV electrocardiogram module databases. Adults presenting with chest pain, dyspnea, syncope, or presyncope and at least 1 ECG within their ED stay were included. Two evaluation cohorts were defined: all stays with ≥1 ECG (n=30,421) and a subset with ≥2 ECGs during the encounter (n=11,273). To predict hospital admission, we first established 2 baseline models: a tabular model (random forest [RF]) trained on structured clinical variables, including demographics, triage acuity, past medical history, medications, and laboratory results, and an ECG-only model that learned directly from raw 12-lead waveforms. We then developed a multimodal deep-learning model that combined ECGs with sequential vital signs as well as the same static tabular features. All models were restricted to data available during the stay up to the time of the last ECG. Performance was assessed with stratified 5-fold cross-validation using identical splits across models.

**Results:**

The multimodal model achieved an area under receiver operating characteristic (AUROC) of 0.911 when trained on all eligible stays. The model predicted disposition after the final ECG was taken, which was a median of 0.3 (IQR 0.2‐5.3) hours after triage and 4.6 (IQR 2.7‐7.3) hours before ED departure. Baseline models performed worse: the ECG-only model had an AUROC of 0.852, and the tabular RF had an AUROC of 0.886. In the subset requiring at least 2 ECGs within the stay, ECG-only reached an AUROC of 0.859, and RF, with the longer interval to chart tabular data, reached a higher AUROC of 0.911. The multimodal model had an AUROC of 0.924 and outperformed baselines in each cohort (paired DeLong *P*<.001).

**Conclusions:**

Serial ECGs, when integrated with evolving vitals and routine clinical features, enable accurate, early prediction of ED disposition in patients presenting with cardiac issues. This open-source, reproducible framework highlights the potential of multimodal deep learning to streamline ED flow, prioritize higher risk cases, and detect evolving, time-critical pathology.

## Introduction

Chest pain, dyspnea, and syncope are among the most common emergency department (ED) chief complaints that eventually result in a cardiac diagnosis [1]. They make up around 16 million encounters yearly in the United States, with chest pain accounting for almost 11 million visits a year [2-4]. Despite their cardiac connotation, the majority prove noncardiac: observational series show that 58.7% of chest pain cases are discharged with a noncardiac diagnosis, adjudication of an international dyspnea cohort found cardiac etiology in 47% and noncardiac causes in the remaining 53% of patients, and only 7%‐10% of syncope presentations to ED are ultimately attributed to a cardiac mechanism [5,6]. However, when a cardiac condition is confirmed, hospitalization becomes far more likely: more than 80% of acute heart failure presentations and up to 86% of high-risk syncope cases are admitted, while admission is far less common for patients whose symptoms are ultimately noncardiac [7,8].

ED providers typically rely on an initial assessment that includes history and physical examination, vital signs, cardiac biomarker tests, and clinical risk scores, such as the emergency severity index (ESI) triage level or the history, electrocardiogram (ECG), age, risk factors, and Troponin (HEART; History, ECG, Age, Risk factors, and Troponin) score [2,9]. However, these traditional risk stratification tools have important limitations. Scores such as ESI and HEART are calculated at a single time point and may not fully reflect evolving patient risk. In practice, initial risk stratification for possible patients presenting with cardiac issues can be insufficient, contributing to ED crowding, which is associated with delayed care, mortality, and generally poorer patient outcomes [10]. This motivates the exploration of advanced machine learning (ML) methods that can integrate multiple data sources and time points to improve predictive performance.

Recent studies have shown that ML and deep-learning models can outperform traditional triage and risk scores in predicting outcomes for patients presenting to the ED. These models often use triage data in combination with vitals, lab results, free-text notes, and past medical history (PMH) to successfully predict general hospitalization or specific critical care outcomes such as acute coronary syndrome [9,11-24]. Fewer recent studies include features within the ED stay, including medications administered, lab tests, and early diagnoses [22-24].

One promising avenue is leveraging deep learning to fuse heterogeneous data sources, including sequential time-series data, such as waveforms, for outcome prediction. Many studies have incorporated ECGs into a disposition model; however, they use implied ECG findings indicated within physician notes or a simple flag indicating whether an ECG was abnormal or conducted [9,15-24]. The implementation of waveforms or more advanced ECG features remains unexplored. Patients with chest pain, dyspnea, presyncope, and syncope often undergo ECGs and vital sign measurements over the course of their ED evaluation. Important prognostic information may lie in the trends and changes in these data. Prior work suggests that sequential data modeling can improve the prediction of patient outcomes. For instance, Bouzid et al [25] demonstrated that analyzing serial ECGs can enhance the detection of acute coronary syndromes: in patients with suspected non-ST-elevation myocardial infarction, combining the prehospital ECG with the initial ED ECG and applying an ML classifier improved diagnostic accuracy and an AI-augmented model further boosted performance to an area under receiver operating characteristic (AUROC) score of 0.83.

Our study builds on these advancements by introducing a multimodal deep-learning approach for early prediction of ED disposition in adult patients presenting with cardiac-related complaints. We developed a multimodal deep-learning model that fuses serial ECG waveforms, sequential vital signs, and key clinical features to predict, in real time, whether a chest-pain patient will require hospital admission. The model will predict disposition after the final ECG available has been taken, requiring anywhere from 1 to 6 ECGs. In contrast to prior works that often focus on diagnostic endpoints or use data available only at presentation, we target the practical outcome of patient disposition and leverage data collected during the ED stay, focusing on ECG waveforms, a novel data stream when predicting ED disposition.

The vast majority of previous studies used private hospital data that were not available for public use [9,12-15,17-24]. The Medical Information Mart for Intensive Care IV (MIMIC IV) is a large deidentified dataset of patients admitted to the ED or an intensive care unit at the Beth Israel Deaconess Medical Center in Boston, MA [26-28]. All data used in this project can be found in the MIMIC-IV, MIMIC-IV Emergency Department module (MIMIC-IV-ED), and MIMIC-IV electrocardiogram module (MIMIC-IV-ECG) modules on PhysioNet [26-30].

The aim of this study was to develop and validate a multimodal deep-learning model that integrates ECG waveforms, sequential vital signs, and tabular clinical data to predict hospital admission in real time. We hypothesized that this fusion of modalities would yield more accurate predictions than conventional methods. If successful, our approach could improve early identification of patients with chest pain, dyspnea, and syncope who require admission (or conversely, those who are safe for early discharge), ultimately enhancing ED decision-making, resource use, and patient outcomes.

## Methods

### Study Cohort

The MIMIC-IV-ED module was filtered to obtain a cohort of 82,907 unique patients with at least 1 mapped ECG from the MIMIC-IV-ECG module within the duration of an ED stay [29,30]. ED stays without ECGs were excluded, and 1 stay per patient was retained.

**Figure 1.**
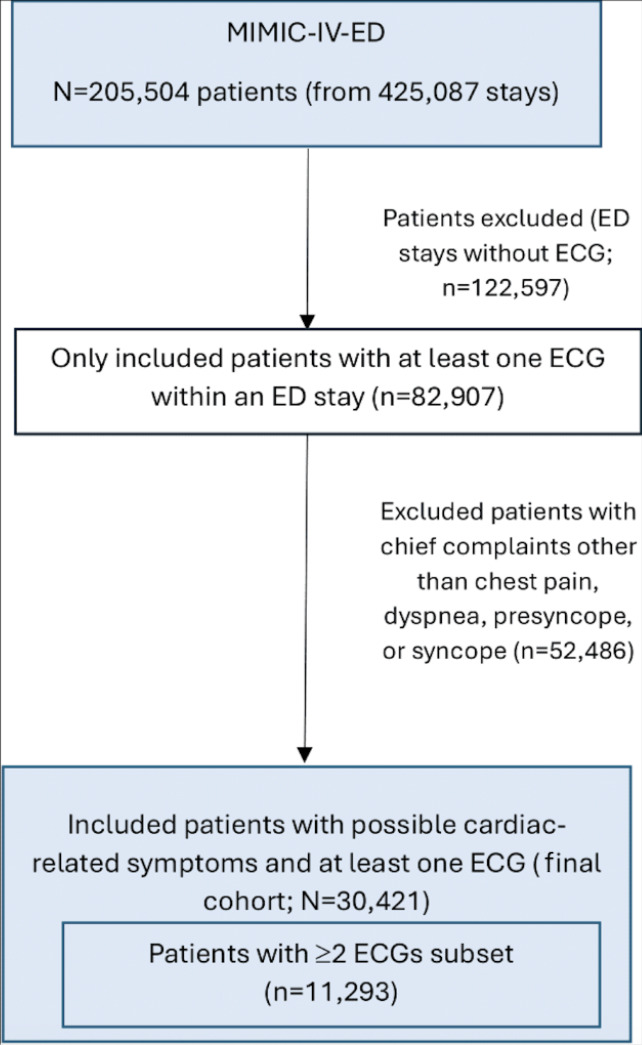
Flowchart of study population. ECG: electrocardiogram; ED: emergency department; MIMIC-IV-ECG: Medical Information Mart for Intensive Care IV electrocardiogram module.

The cohort was then filtered only to include patients whose chief complaints contained keywords relating to presyncope, syncope, dyspnea, and chest pain. After filtering patients with a disposition other than discharge or admission, 30,421 unique patients presenting to the ED with possible cardiac-related symptoms and at least 1 ECG were left in the study (Figure 1). The number of ECGs per stay and the length of stay before the final ECG from which the model predicts disposition were additionally noted as static features.

### Feature Extraction

#### ED Lab Results and Medications (Static)

From MIMIC-IV hospital data, we extracted ED labs and medications recorded after ED arrival and before the final ECG. The lab results included were Troponin T, creatinine, lactate, C-reactive protein, B-type natriuretic peptide, hemoglobin, potassium, magnesium, and white blood cell count. For each lab, we derived 4 features: first value, peak value, an abnormal flag, and a “missing” flag. ED medications were identified through Pyxis Generic Sequence Number codes, which were mapped to Enhanced Therapeutic Classification codes and grouped by their first 6 digits. Each group contributed a binary feature (38 features). All lab tests and medications were incorporated as static features and were not modeled sequentially or imputed, since both missingness and sequence length varied substantially across patients, making static summarization the most consistent approach.

#### PMH (Static)

We included the disposition of the prior visit and the number of previous stays visible in the MIMIC-IV database. Prior *International Classification of Diseases* diagnoses from a MIMIC hospital visit were binned to Clinical Classifications Software for *International Classification of Diseases, 9th Revision* and Clinical Classifications Software Redefined for *International Statistical Classification of Diseases and Related Health Problems 10th Revision* categories (253 variables). Outpatient prescriptions in the past year were binned to 7 broad Enhanced Therapeutic Classification groups.

#### Other Static Features

Age, sex, and acuity were included along with arrival mode and chief complaint Boolean operators. We added total counts for ED medications, prior medications, and prior diagnoses.

#### Vitals (Sequential)

We incorporated temperature, heart rate, respiratory rate, oxygen saturation, systolic and diastolic blood pressure, and pain recorded before the final ECG. All vitals taken at triage were included, as well as vitals drawn throughout the ED stay, with chart time before the last ECG for that stay. All vitals except for pain were nonmissing at triage but contained a large number of missing values when later charted during the stay. Missing vitals, excluding pain, were imputed using a Bayesian Regression formula fit on the training set per fold. Missing flags were created for each vital to note whether the vital was initially missing before imputation. Two additional columns were added, stating whether the patient was sleeping or unable to answer based on the string value of pain.

#### ECG Feature Extraction

In order to best implement ECGs for the downstream task of predicting hospitalization, an AI model was used to encode a lower dimensional ECG feature vector. Specifically, a one-dimensional residual neural network model (ResNet-18) was chosen due to its ability to learn discriminative temporal and spatial features from high-dimensional ECG signals while avoiding vanishing gradients through skip connections. The network takes in an ECG with 5000 samples (500 Hz for 10 seconds) for each of the 12 leads and outputs one singular 512-dimensional feature vector. In the first approach, the ResNet-18 was initialized for hospitalization prediction with random weights. In the second, it was first trained on a larger ECG dataset with labels unrelated to ED disposition, to capture general waveform structure and patterns. Training beforehand allows the network to initialize with physiologically relevant weights, enhancing feature extraction of QRS-T morphology and inter-lead patterns, while significantly reducing overfitting through faster convergence and freedom to freeze layers. Reduced overfitting may be important given the added complexity of the multimodal architecture.

**Figure 2.**
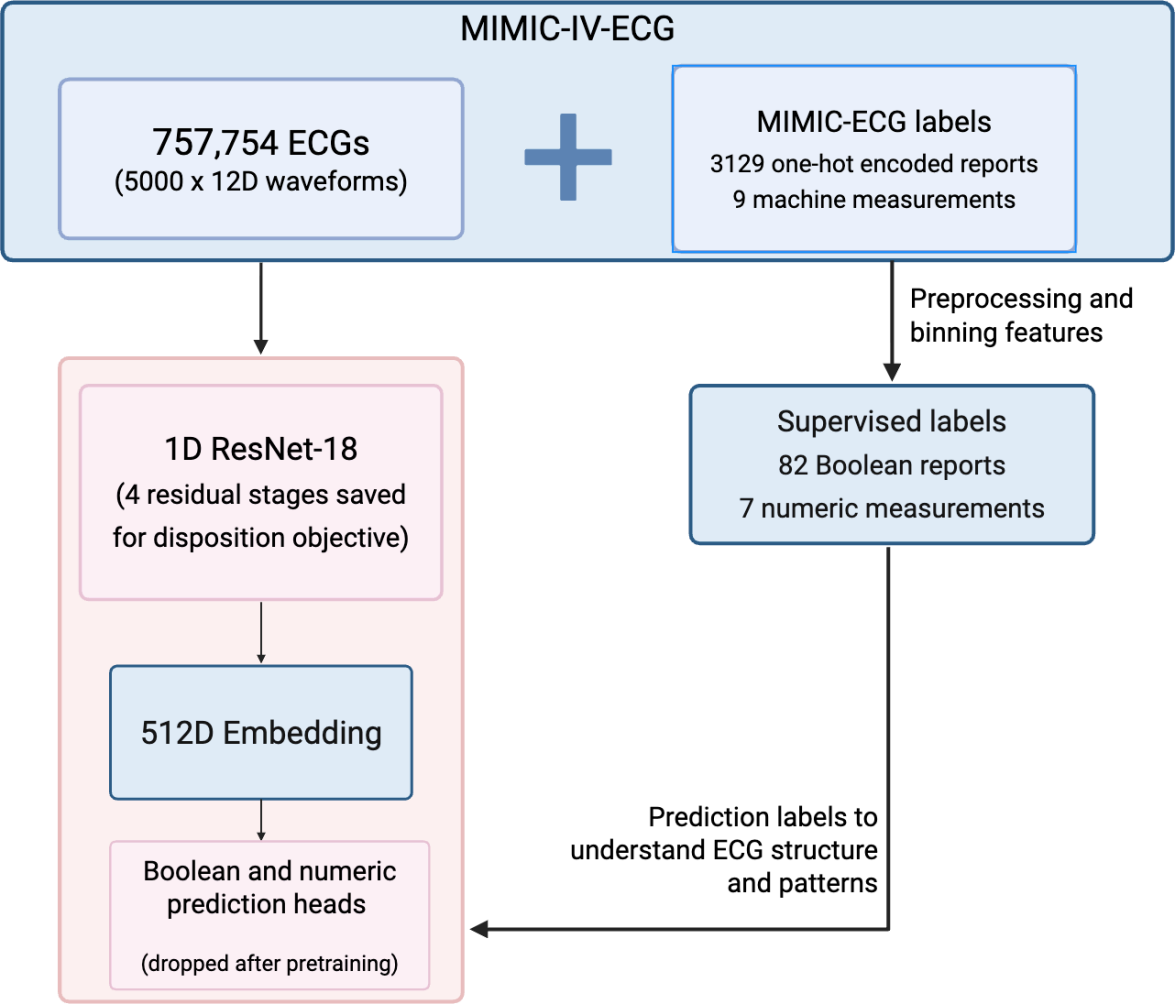
Flowchart of supervised pretraining workflow for the transfer-learning approach. D: dimensional; ECG: electrocardiogram; MIMIC-IV-ECG: Medical Information Mart for Intensive Care IV electrocardiogram module; ResNet-18: residual network model.

As shown in Figure 2, the full MIMIC-IV-ECG dataset was used as a means of supervised pretraining, in which each ECG waveform was used as an input to predict MIMIC machine measurements and machine-generated reports. These measurements and reports are provided on the MIMIC-IV-ECG module for every available ECG. Machine measurements reflect quantitative ECG characteristics across all leads, including the average RR interval, QRS axis, T-axis, and several more. Machine-generated reports are stored as strings in columns labeled report 1 through report 17, with example strings being “Atrial Fibrillation,” “ST-elevation,” or “Normal ECG.” After one-hot encoding, 3129 columns were initially created. After removing labels that had too few positive instances, along with combining truth values of columns with slight syntax variance or similar clinical significance, such as “ventricular pacing” and “ventricular-paced rhythm,” 82 unique Boolean features remained.

Before training, all ECGs were cleaned using the Neurokit2 signal processing library [31]. A one-dimensional ResNet-18 model was trained to predict both numeric and Boolean features corresponding to each of the total 7,57,754 ECGs. Mean squared error was used to evaluate the loss of numeric features (machine measurements), and binary cross-entropy (BCE) was used to evaluate the loss of Boolean features (machine-generated reports). The total loss function represented the mean squared error loss added to the BCE loss multiplied by 9 (due to far more Boolean labels than numeric).

### Prediction Model

#### Multimodal

A multimodal model trained on sequential ECG waveforms, sequential vitals data, and 353 static variables was built to predict hospitalization in patients presenting to the ED. The model used all data gathered during the stay before the final ECG. The model was trained on all stays as well as on a subset of stays containing at least 2 ECGs to quantify the importance of sequential ECGs and more available data within the time window.

Raw 12-lead ECGs were first cleaned with NeuroKit2 and subsequently one-dimensional ResNet-18 initialized with random weights or the ResNet-18 that had been trained on the larger MIMIC-IV-ECG dataset (757,754 recordings). In the transfer-learning approach, ResNet-18 layers through block layer3 were frozen: only layer4 and the linear adapter were trained during finetuning. The final global-average-pooling layer of the backbone was replaced with a linear adapter so that each waveform was mapped to a fixed-length, 512-dimensional embedding.

**Figure 3.**
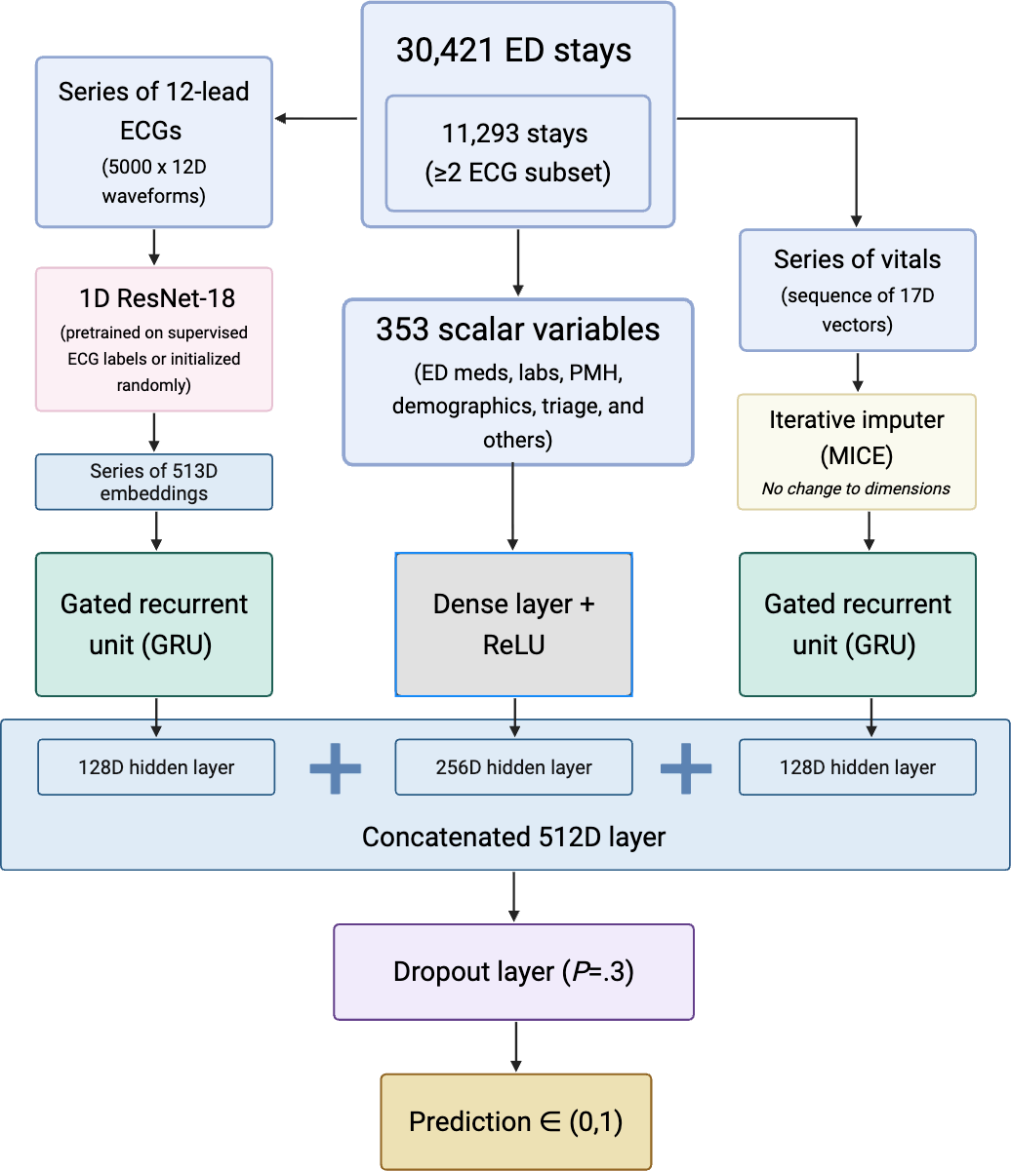
Diagram of the multimodal model architecture approach to predict hospitalization in the full and subset cohorts. D: dimensional; ED: emergency department; PMH: past medical history; ReLU: rectified linear unit; ResNet: residual network model.

For every ED stay we retained, in temporal order, all ECGs recorded before the clinical disposition decision (maximum 6 per stay) and all vital sign rows charted before the last ECG (maximum 10 per stay). The ECG and vital embeddings were first stacked and padded to match the maximum length. As shown in Figure 3, 2 independent, single-layer gated recurrent units (GRUs) were used to summarize these sequences. Before being passed to the GRU, padded batches were converted to PackedSequence objects, ensuring the recurrent unit was unrolled only over the valid timesteps and ignored the artificial zero-padding. The time before the final ECG was added as an extra time-delta channel in the embeddings of each ECG and vitals sequence. The ECG GRU read the sequence of 513-dimensional embeddings and returned a 128-dimensional hidden state h_ECG_; the vitals GRU processed a 17-variable vector at each timestep—7 *z*-scored physiological values, 1 time-delta channel, and 9 binary mask indicators denoting whether the original measurement had been missing, as well as unable or sleeping for “pain”—and produced a similar 128-dimensional summary h_VITALS_.

All 353 static features were concatenated, normalized with the mean (SD) calculated on the training set, and projected by a fully connected layer with rectified linear unit activation to a 256-dimensional vector *s*.

The 3 modality-specific representations were then fused through simple concatenation,

*z* = [ h_ECG_|| h_VITALS_ || *s* ] ℝ^512^ , followed by an additional dropout layer (rate=0.30) and a single neuron that yielded the estimated probability of hospital admission p̂ ∈ (0,1).

Model parameters introduced during this study were optimized with AdamW (weight decay 10^–4^ at a learning rate of 3 × 10^–4^ [32]; the adapter atop the ECG encoder was updated at 10^–4^. BCE with class weighting was used as the optimization objective.

#### Baseline

To measure the effect of ECGs alone in predicting disposition, separate models were trained on only ECG features. The ECG-only model used the same parameters and architecture as the multimodal ECG branch.

Random forest (RF) is an ML algorithm that combines predictions from an ensemble of decision trees to produce 1 final classification. An RF was constructed solely on the 353 static tabular features as a baseline to the multimodal architecture. Both the ECG-only and tabular baseline models were evaluated in the full cohort (“all stays”) and in the ≥2-ECG subset using the exact stratified 5-fold cross-validation splits from the multimodal pipeline.

#### Model Evaluation

Three models (multimodal, tabular, and ECG-only) were evaluated on the full cohort of all stays and on the ≥2-ECG subset. Across cross-validation folds, the mean AUROC, area under the precision-recall curve, *F*_1_-score, precision, and recall were reported. Thresholds for *F*_1_-score, precision, and recall were selected using a nested procedure that maximized *F*_1_-score on training folds and applied it to the validation fold. Pairwise AUROC differences between models were tested with the DeLong method.

### Ethical Considerations

This study used deidentified data from the PhysioNet MIMIC-IV database under a data use agreement. Because the data are fully deidentified, the study was determined to be exempt from institutional review board (IRB) review, and informed consent was waived in accordance with applicable regulations. All data handling complied with relevant privacy and data protection standards, and no identifiable personal information was accessed. No participant contact or compensation occurred. This research was conducted in accordance with the ethical standards of the institutional and national research committees and with the 1975 Declaration of Helsinki (as revised in 2000).

## Results

### ED Timing Analysis

Of the 30,421 ED stays included in our analysis, 11,273 (37.1%) involved stays with ≥2 ECGs (Table 1). In the full cohort, the model issued its prediction a median of 0.3 (IQR 0.2‐5.3) hours after triage, leaving a median of 4.6 (IQR 2.7‐7.3) hours before the patient physically left the ED. Note that these medians are calculated independently and therefore do not sum to the overall median length of stay. As a result, disposition was predicted in the first 18 minutes for most visits, but for the upper quartile, the model leveraged data gathered more than 5 hours into the encounter. In the ≥2 ECG subset, the model predicted disposition far later, with a median of 6.5 (IQR 4.3‐9.8) hours after triage. The median time from prediction to disposition was also shorter than the full cohort, at 3.4 (IQR 1.7‐9.1) hours. However, the 75th-percentile (Q3) prediction lead time exceeded 9 hours, meaning the model anticipated disposition more than 9 hours before the actual decision. This subset also had a longer overall ED length of stay (median 9.5, IQR 6.9‐17.8 hours vs 6.5, IQR 4.3‐9.8 hours in the full cohort). Because the ≥2 ECG cohort had more ECGs taken and the model predicts disposition after the final ECG, it often took more time to reach the final ECG.

**Table 1.** Baseline characteristics and measurements in all versus ≥2-ECG^a^ cohorts.

Metrics^b^	All stays (N=30,421)	≥2 ECG stays (n=11,273)
Admit, n (%)	13,138 (43.2)	4070 (36.1)
Acuity, mean (SD)	2.3 (0.6)	2.2 (0.6)
Age (years), mean (SD)	57.1 (19.8)	61.7 (16.3)
ECG metrics		
Number of ECGs, mean (SD)	1.48 (0.74)	2.30 (0.64)
≥2 ECGs, n (%)	11,273 (37.1)	11,273 (100)
≥3 ECGs, n (%)	2538 (8.3)	2538 (22.5)
LOS^c^ after final ECG (hours), median (IQR)	4.6 (2.7‐7.3)	3.4 (1.7‐9.1)
LOS before final ECG (hours), median (IQR)	0.3 (0.2‐5.3)	6.5 (3.9‐7.7)
Full LOS (hours), median (IQR)	6.5 (4.3‐9.8)	9.5 (6.9‐17.8)
Laboratory values (first)		
Troponin T (ng/mL), median (IQR)	0.1 (0‐0.2)	0.1 (0‐0.2)
Missing, n (%)	28,376 (93.3)	9579 (85)
Lactate (mmol/L), median (IQR)	1.7 (1.3‐2.3)	1.7 (1.3‐2.3)
Missing, n (%)	27,856 (91.6)	9206 (81.7)
Creatinine (mg/dL), median (IQR)	0.9 (0.8‐1.1)	0.9 (0.8‐1.1)
Missing, n (%)	18,327 (60.2)	1177 (10.4)
B-type natriuretic peptide (pg/mL), median (IQR)	1092.5 (180‐4555.8)	948.5 (162‐4293.2)
Missing, n (%)	27,993 (92)	9363 (83.1)
Potassium (mmol/L), median (IQR)	4.2 (3.9‐4.6)	4.2 (3.9‐4.6)
Missing, n (%)	18,340 (60.3)	1170 (10.4)
Magnesium (mg/dL), median (IQR)	2 (1.9‐2.2)	2 (1.9‐2.2)
Missing, n (%)	26,702 (87.8)	8244 (73.1)
Presenting symptoms, n (%)		
Chest pain	17,898 (58.8)	8519 (75.6)
Dyspnea	9477 (31.2)	2575 (22.8)
Syncope	4924 (16.2)	983 (8.7)
Summary counts, n (%)		
No visible ED^d^ medications before final ECG	21,368 (70.2)	3583 (31.8)
No visible prior medications (past year)	22,074 (72.6)	8157 (72.4)
No visible prior hospital diagnosis	15,950 (52.4)	5275 (46.8)
No visible prior ED stay	16,594 (54.5)	5591 (49.6)

a ECG: electrocardiogram.

b Percentages for all rows are calculated using unique patients as the denominator (N). The values from the selected representative lab measurements are from tested patients only.

c LOS: length of stay.

d ED: emergency department.

Detailed vitals summaries, including missingness counts by cohort, are shown in Table S1 in Multimedia Appendix 1.

### Missing Values

When vitals were charted, only temperature was frequently missing within 41.8% (28,573/68,371) of vital chartings within all stays and 55% (25,376/46,098) in the ≥2 ECG subset (Table S1 in Multimedia Appendix 1). All other vitals were <7% missing per charting, and many stays had multiple vitals chartings before the prediction cutoff. Of the 9 lab values this study accounted for, 6 were entirely missing in more than 90% (~27,400/30,421) of all patients before the prediction cutoff (Table 1). Because the subset included a longer median interval before the prediction cutoff, the proportion of missing laboratory results fell for every test. Notably, creatinine dropped from 60.2% (18,327/30,421) to 10.4% (1177/11,273), missing between the cohorts. PMH within the dataset’s visibility was very limited. Any prior diagnoses and ED stays were each completely missing in more than half of the total patients. A total of 72.6% (22,074/30,421) of patients had no visible medications prescribed to them in the past year in the dataset.

### ECG Feature Extractor

Compared with random initialization, supervised transfer learning of the ResNet-18 did not yield statistically significant improvements in predictive performance. However, supervised pretrained models consistently converged faster and achieved marginally higher metrics in the ≥2 ECG subset. Accordingly, all reported results used the transfer-learning approach.

### Model Evaluation

Using a partially frozen pretrained ResNet-18 as an ECG encoder, a multimodal dual GRU fusion net was trained on the full cohort and ≥2 ECG subset to predict hospital admission. For comparison, an ECG-only variant (same encoder and GRU using only raw 12-lead waveforms) and a tabular-only RF were trained for the same task. Performance is shown in Table 2.

**Table 2.** Performance metrics for ECG^a^-only, multimodal, and tabular random forest models. Evaluations use an identical time cutoff across models and are stratified by cohort (all stays; ≥2 ECGs). Expected calibration error was computed from 10 quantile bins. *P* values are from paired DeLong tests versus the multimodal model. Metrics are mean values across the 5-fold cross-validation.

Models	AUROC^b^, mean (SD)	AUPRC^c^, mean (SD)	Precision, mean (SD)	Recall, mean (SD)	ECE^d^, mean (SD)	*P* value
All stays						
ECG only	0.852 (0.003)	0.813 (0.005)	0.698 (0.034)	0.803 (0.036)	0.034 (0.018)	<.001
Tabular (RF^e^)	0.886 (0.003)	0.849 (0.006)	0.745 (0.017)	0.830 (0.014)	0.024 (0.004)	<.001
Multimodal	0.911 (0.004)	0.889 (0.005)	0.784 (0.041)	0.839 (0.046)	0.026 (0.008)	—^f^
≥2 ECG stays						
ECG only	0.859 (0.011)	0.794 (0.017)	0.674 (0.029)	0.760 (0.038)	0.053 (0.018)	<.001
Tabular (RF)	0.911 (0.006)	0.865 (0.014)	0.774 (0.016)	0.813 (0.013)	0.039 (0.008)	<.001
Multimodal	0.924 (0.009)	0.889 (0.016)	0.807 (0.030)	0.808 (0.024)	0.040 (0.024)	—

a ECG: electrocardiogram.

b AUROC: area under receiver operating characteristic.

c AUPRC: area under the precision-recall curve.

d ECE: expected calibration error.

e RF: random forest.

f Not applicable.

The multimodal model in the multiple ECG subset yielded the highest metrics, with an AUROC of 0.924. The tabular RF model yielded slightly lower metrics (AUROC=0.91). Across cohorts, the AUROC of the ECG-only model differed by as little as AUROC of 0.007, suggesting that additional information from prior ECGs in the same stay does not improve prediction by a significant margin. In the all-stays cohort, where predictions were made much earlier in the ED course, the performance gap between the random forest and multimodal models was larger: AUROC 0.886 versus 0.911 (*P*<.001). These findings indicate that combining ECG waveforms with vitals provides the largest benefit when static tabular data, such as lab results, are sparse early in the encounter.

### Static Feature Importance

**Figure 4.**
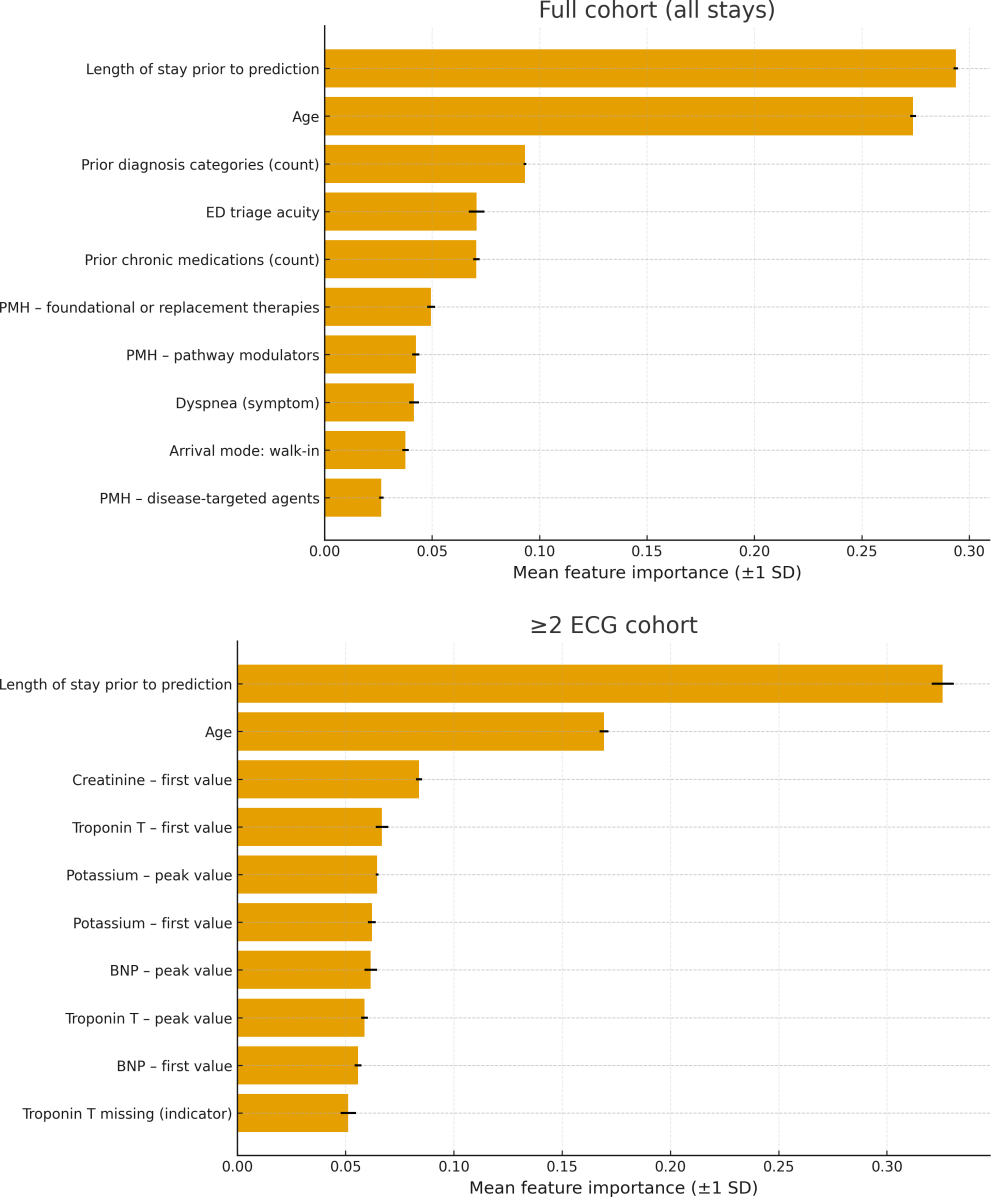
Two bar graphs showing mean feature importance across 5 folds for both cohorts. BNP: B-type natriuretic peptide; ECG: electrocardiogram; ED: emergency department; PMH: past medical history.

The 10 static features with the highest mean feature importance in both cohorts for the tabular RF model are displayed in Figure 4. All 4 features corresponding to Troponin were included in the top 10 in the multiple ECG subset, but none appear in the full cohort of all stays. The greater importance of lab values in the multiple ECG subset is most likely due to less missing lab data, as the median cutoff for prediction later. The length of stay prior to prediction and the patient’s age remain the most important features within both cohorts. In the full cohort, PMH and acuity were given more importance.

We also conducted an ablation study evaluating a tabular RF trained on only the top 10 static features (vs all 353). On the multi-ECG subset, the reduced model achieved an AUROC of 0.862 compared to 0.911 with the full feature set. On the full cohort, performance decreased from 0.886 to 0.841 when limited to 10 features.

## Discussion

### Principal Findings

In this study, we developed a multimodal deep-learning model that integrates sequential ECG waveforms and vital signs, along with static tabular features, to predict hospitalization in real time for patients presenting to the ED with possible cardiac presentations. Our results show that ECGs can also be used alone to predict disposition, but are best used in combination with vitals and other commonly implemented static features such as lab results, medications administered, prior medical history, and triage data. The primary operational value of multimodal fusion appears early when tabular data are sparse, whereas later in the visit, a simpler tabular model may achieve comparable discrimination at lower implementation cost.

The multimodal model that required at least 2 ECGs achieved the highest performance of AUROC of 0.924 in predicting hospitalization. This model predicted a disposition of 3.4 (IQR 1.7‐9.1) hours in advance on the median, but more than 9 (median 9.5, IQR 6.9‐17.8) hours in advance for the top 25th percentile with high accuracy. However, a median of 6.5 (IQR 3.9‐7.7) hours was spent in the ED before this model reached a prediction. The RF built from tabular data achieved a slightly lower AUROC of 0.911. As the ECG-only model’s performance did not improve significantly with more ECGs, the higher AUROC in the ≥2 ECG subset for the multimodal and RF models is most likely due to the more lenient cutoff to chart lab results and ED medications before prediction.

When trained on all-stays (median prediction time less than 20 minutes after triage), the multimodal architecture yielded significantly higher metrics in comparison to tabular and ECG-only models. Incorporating additional data streams such as waveforms and vitals can lead to better and more reliable hospitalization predictions when traditional clinical data are sparse.

### Comparison With Prior Work

Our ECG-based prediction models substantially outperform conventional triage tools such as the nurse-assigned ESI level, which typically achieves AUROCs in the 0.69‐0.70 range and is at par with or better than many prior ML models that used only triage-time data [11]. For example, Raita et al [11] reported an AUROC of 0.82 for a deep neural network predicting hospitalization using initial-only triage information, and Hong et al [12] similarly found approximately 0.87 using triage data with extreme gradient boosting and a neural network. In addition, Hong et al [12] also created extreme gradient boosting and deep neural network models, which reached an AUROC of 0.92, implementing PMH and triage data to predict hospital admission at the beginning of the ED stay. Their approach is further discussed in the limitations section of this study.

Some studies make use of data after triage to predict disposition. Barak-Corren et al [24] achieved high performance (AUROC=0.97 within 1 h of triage) using logistic regression on standard demographic, triage, medication, lab, vital, and PMH data in a single-site Israeli ED. However, they train their logistic regression model to predict disposition on a per-visit basis, while we provide a per-patient approach, a more challenging task because there is no patient overlap within the train and validation cohort. Sezik et al [22] used similar data (lab results, history, and triage variables) in combination with vitals and other features extracted from text to reach an AUROC of 0.960 with an RF classifier. However, they do not incorporate a cutoff time and use features conducted throughout the entire ED stay, including the length of stay. In contrast, our study stops feature collection after a given ECG in order to predict later changes in a patient presenting with a cardiac condition and provide an early hospitalization prediction as opposed to serving as an aid to decide disposition at the end of the stay.

Importantly, our approach maintained high accuracy despite the broad outcome of general hospital admission, which includes many noncritical cases; this is notable because prior ML studies in chest pain often focused on narrower critical events. For instance, a recent study targeted only critical care outcomes such as ED cardiac arrest or intensive care unit transfer and achieved an AUROC of ~0.95 with a tailored Least Absolute Shrinkage and Selection Operator logistic model [13]. The fact that we still attained strong performance (AUROC=0.911 and 0.924) for a broader outcome of hospitalization for any reason underlines the effectiveness of our multimodal fusion strategy. Using the MIMIC-IV-ED and MIMIC-IV-ECG modules (which include more than 400,000 ED stays and 800,000 ECGs, respectively) ensured that our work is widely reproducible [29,30]. This reproducibility and open-source approach contrasts with many prior ED prediction studies that relied on proprietary data not available to outside researchers [9,12-15,17-24]. By creating our model from a public database, we demonstrate the feasibility of developing advanced prediction tools using open data.

### Limitations and Future Work

This study has several limitations. First, all data used to train the model are specific to the single-center MIMIC-IV database from the Beth Israel Deaconess Medical Center. This limits generalizability, as the model may overfit to site-specific patterns in the high-dimensional input space. Thus, external validation is needed. Nonetheless, much of the data used, such as common 12-lead ECGs, vitals, and standardized eHealth records, could be readily translated in future multicenter studies to support broader applicability.

Second, due to exploring the promise of ECG waveforms on ED disposition, our study relies on the patient presenting with a possible cardiac condition, which in this case only includes patients presenting with chest pain, dyspnea, presyncope, and syncope. These symptoms were chosen as they are the most common in the ED and are predominantly cardiac in origin, in contrast to less frequent and more heterogeneous complaints such as palpitations. In addition, all included patients received at least 1 ECG, and although it is standard for patients with possible cardiac symptoms to promptly receive an ECG, this could still affect the model’s generalizability. Future work should test whether expanding the cohort further broadens the model’s applicability.

Third, many variables were largely missing, including more than 90% of most labs tested and 41% to 55% of temperature readings when vitals were charted. As opposed to the 0.92 AUROC neural network proposed by Hong et al [12], which contained full patient medical records and a comprehensive PMH of each patient, we only included past history visible within the MIMIC-IV and MIMIC-IV-ED modules. Overall, 72.6% (22,074/30,421) of patients in our study set had no prior medications, 52.4% (15,950/30,421) had no prior diagnoses, and 54.5% (16,594/30,421) had no prior ED stays. PMH recorded at another hospital was not visible. Considering the value shown by incorporating a comprehensive PMH in predicting disposition, our model performance could significantly benefit from having access to more prior diagnoses, medications, and ED stays.

Finally, like most disposition models, our model treats the disposition recommended by the ED provider as a truth label. Thus, our model may be, to some extent, learning institutional decision policies that can vary across hospitals in addition to patient physiology. The fact that “Length of stay prior to prediction” was the most predictive static variable likely reflects a confounding effect. Longer ED stays often indicate physician uncertainty or patient complexity, meaning the model may capture care processes as well as outcomes. Although discrimination was highest in the ≥2-ECG subset, its prediction was issued a median of 6.5 (IQR 4.3‐9.8) hours after triage (~3.4 hours before ED departure), which may limit clinical use for throughput and flow optimization.

In practice, the model would run automatically after each ECG, showing a calibrated admission probability and risk band in the EHR as a banner alert. The model could also be rerun with new vitals or lab measurements, which are automatically entered into the patient’s record. At inference, the system runs only forward passes through a 1D ResNet-18 encoder and small GRUs, making it suitable for near-real-time usage. We envision it as decision support that complements existing scores early in the visit, with a tiered strategy where a simpler tabular model can suffice later, once more labs and medications are available. Future work should explore including more data modalities in a time-series format and possibly leaving the cutoff for prediction variable throughout updates in lab testing, vitals, and diagnostic tools such as an ECG. Incorporating novel data streams, such as the ECG with a more complete PMH, could lead to a far better prediction as well. Improving model interpretability, especially within deep-learning models, is also crucial to gain clinician trust and improve decision-making in the ED.

### Conclusions

We developed and validated a multimodal deep learning model that combines ECG waveforms, vital signs, and static clinical features to predict hospital admission in patients presenting to the ED with potential cardiac complaints. The model achieved a higher AUROC than both ECG-only and tabular-only baselines and issued predictions at the time of the final ECG, a median of 0.3 (IQR 0.2‐5.3) hours after triage. These findings suggest that integrating ECG waveforms with sequential and static data may enhance early risk stratification in the tested patient population. Future external validation is required to assess generalizability.

## Supplementary material

10.2196/80569 Multimedia Appendix 1 Vitals measurements in all versus ≥2 electrocardiogram cohorts.

## Abbreviations

AUROC: area under the receiver operating characteristic curve
BCE: binary cross-entropy
ED: emergency department
ESI: emergency severity index
GRU: gated recurrent unit
HEART: History, ECG, Age, Risk factors, and Troponin
MIMIC-IV: Medical Information Mart for Intensive Care IV
MIMIC-IV-ECG: MIMIC-IV electrocardiogram module
MIMIC-IV-ED: MIMIC-IV Emergency Department module
ML: machine learning
PMH: past medical history
ResNet-18: residual neural network
RF: random forest
