# Supplementary material for: Serial 12-Lead Electrocardiogram–Based Deep-Learning Model for Hospital Admission Prediction in Emergency Department Cardiac Presentations: Retrospective Cohort Study
Source: JMIR Cardio. 2025 Oct 17;9:e80569. doi: 10.2196/80569 (PMC12533930; doi:10.2196/80569)
Supplement: Multimedia Appendix 1 [file cardio-v9-e80569-s001.docx]

**Table S1.**

| **Vitals** | **N=68,371** | **N=46,098** |
| --- | --- | --- |
| Temperature, °F, median (IQR) | 98.0 (97.6–98.4) | 98.0 (97.7–98.4) |
| Missing, n (%) | 28,573 (41.8%) | 25,376 (55.0%) |
| Heart rate, bpm, median (IQR) | 78.0 (67.0–90.0) | 75.0 (65.0–88.0) |
| Missing, n (%) | 2,431 (3.6%) | 1,618 (3.5%) |
| Respiratory rate, breaths/min, median (IQR) | 18.0 (16.0–19.0) | 18.0 (16.0–19.0) |
| Missing, n (%) | 3,338 (4.9%) | 2,341 (5.1%) |
| Oxygen saturation, %, median (IQR) | 99.0 (97.0–100.0) | 98.0 (97.0–100.0) |
| Missing, n (%) | 4,661 (6.8%) | 3,628 (7.9%) |
| Systolic BP, mm Hg, median (IQR) | 131.0 (117.0–147.0) | 130.0 (116.0–146.0) |
| Missing, n (%) | 2,970 (4.3%) | 1,994 (4.3%) |
| Diastolic BP, mm Hg, median (IQR) | 75.0 (65.0–85.0) | 74.0 (64.0–84.0) |
| Missing, n (%) | 3,027 (4.4%) | 2,016 (4.4%) |

^a^Abbreviations: BP, blood pressure; SBP, systolic blood pressure; DBP, diastolic blood pressure.

^b^Percentages for missing vitals are calculated using total charted measurements, not unique patients (one patient may have more than one set of vital sign measurements charted before their final ECG).
